# Possible neuroprotective role of P2X2 in the retina of diabetic rats

**DOI:** 10.1186/s13098-018-0332-7

**Published:** 2018-04-12

**Authors:** Jorge E. Mancini, Gustavo Ortiz, Constanza Potilinstki, Juan P. Salica, Emiliano S. Lopez, J. Oscar Croxatto, Juan E. Gallo

**Affiliations:** 1 Department of Ophthalmology, Nanomedicine & Vision Group, Facultad de Ciencias Biomédicas, Universidad Austral, Av. Juan D. Perón 1500, B1629AHJ Pilar, Buenos Aires Argentina; 2 Department of Ocular Pathology, Fundación Oftalmlógica Argentina “Jorge Malbran”, Buenos Aires, Argentina; 3 Instituto de Investigaciones en Medicina Traslacional (IIMT), Universidad Austral, Consejo Nacional de Investigaciones Científicas y Técnicas (UA-CONICET), Pilar, Buenos Aires, Argentina

**Keywords:** Diabetic retinopathy, P2X2, PPADS, Retina, VEGF-A

## Abstract

**Background:**

Purinergic receptors are expressed in different tissues including the retina. These receptors are involved in processes like cell growth, proliferation, activation and survival. ATP is the major activator of P2 receptors. In diabetes, there is a constant ATP production and this rise of ATP leads to a persistent activation of purinergic receptors. Antagonists of these receptors are used to evaluate their inhibition effects. Recently, the P2X2 has been reported to have a neuroprotective role.

**Methods:**

We carried out a study in groups of diabetic and non-diabetic rats (N = 5) treated with intraperitoneal injections of PPADS, at 9 and 24 weeks of diabetes. Control group received only the buffer. Animals were euthanized at 34 weeks of diabetes or at a matching age. Rat retinas were analyzed with immunohistochemistry and western blot using antibodies against GFAP, P2X2, P2Y2 and VEGF-A.

**Results:**

Diabetic animals treated with PPADS disclosed a much more extended staining of VEGF-A than diabetics without treatment. A lower protein expression of VEGF-A was found at the retina of diabetic animals without treatment of purinergic antagonists compared to diabetics with the antagonist treatment. Inhibition of P2X2 receptor by PPADS decreases cell death in the diabetic rat retina.

**Conclusion:**

Results might be useful for better understanding the pathophysiology of diabetic retinopathy.

## Background

The P2 receptors are divided in two groups: P2X and P2Y receptors. P2X receptors are cation-selective channels with almost equal permeability to Na^+^ and K^+^, and significant permeability to Ca^2+^. They gate extracellular cationic response to ATP and are widely expressed in the central nervous system and in the body periphery, where they play important roles in different processes, including muscle contraction, modulation of the cardiovascular and respiratory systems, and transmitter release [1]. P2Y receptors are G-protein coupled receptors that are divided into two subfamilies. The first family is composed of P2Y receptors 1, 2, 4, 6, and 11 that predominantly couple to Gq, thereby activating phospholipase C, which results in mobilization of intracellular Ca^2+^. The second family consists of P2Y receptors 12, 13, and 14, which are G-coupled that inhibit adenyl-cyclase and regulate ion channels. The P2Y receptors account for broad and varied physiological responses such as platelet aggregation, granulocyte differentiation, and regulation of vascular tone [2, 3].

P2X and P2Y receptor expression in the rat retina has been reported before [4–6]. Recent studies have suggested a role for P2 receptors in retinal disease, including retinal detachment [7, 8], proliferative vitreoretinopathy [9, 10], diabetic retinopathy [11–14], retinal degeneration [15, 16] and oxygen-induced retinopathy [17]. Purinergic receptors are involved in chronic inflammation [18] and their upregulation has been seen in diabetic complications [19]. Diabetic retinopathy is known to be, at least in part, an inflammatory disease. However, the role of purinergic P2 receptors in the inflammatory mechanism of diabetic retinopathy (DR) has been scarcely investigated. Moreover, there are very few studies on P2X2 and P2Y2 receptors in the disease.

The P2Y2 receptor is activated by ATP and UTP. Different functional studies have shown that P2Y2 receptors are present in endothelial cells and fibroblasts [20–24], glial cells [25–27], pancreatic cells [28–30], and pituitary cells [1]. Purinergic signaling seems to be a mediator between the retina and the RPE [31]. The P2X2 and P2Y2 have been reported to play a neuroprotective role, particularly in neuronal survival [32, 33].

Taking into account that purinergic receptors are expressed in the rat retina, their participation in chronic inflammation as well as in degenerative, metabolic and neuroprotective process we thought it was interesting to carry out a study on the role of P2X2 and P2Y2 in the diabetic retina. For this purpose, we performed a histological and immunohistochemical analysis in diabetic and non-diabetic rats, using pyridoxalphosphate-6-azophenyl-2′,4′-disulfonic acid (PPADS), a non-specific P2X2 and P2Y2-like antagonist. We have also analyzed the neurotrophic and pro-inflammatory molecule VEGF-A considering its interaction with components of the purinergic pathway and its role in the development of diabetic retinopathy and in the neuroprotection of the ischemic injury [34, 35].

## Methods

### Animal model

**Fig. 1:**
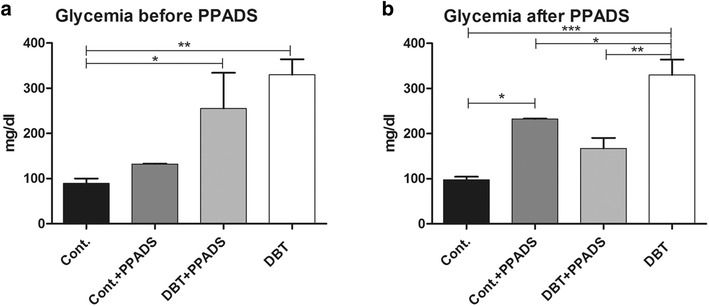
Fast glycemia. The two bar charts show the analyses of fast glycemia in two different periods in the seven groups of animals. **a** Glycemia levels at 9 weeks after vehicle or streptozotocin treatment in control and diabetic animals. Measurement was done 1 day before PPADS first cycle treatment. All control groups had normal glycemia levels before purinergic receptors antagonist treatment. **b** Glycemia levels at 34 weeks after two purinergic receptors antagonist treatment cycles performed at 9 and 24 weeks of diabetes. *Cont.* control; *PPADS* pyridoxalphosphate-6-azophenyl-2′,4′-disulfonic acid; *DBT* diabetic

Five-month-old Sprage-Dawley male rats were used in this study. Animals were kept at constant 12 h/12 h light dark cycle with food and water *ad libitum*. Diabetes was induced by an intraperitoneal (IP) dose of streptozotocin (STZ) (45 mg/kg) in 100 µL of a 0.1 M solution of citrate buffer of 154 mL of NaCl at 4.5 pH [36]. Twenty-four hours later diabetes condition was verified by a measurement of fast blood glucose with a tail snipping using a sample of 32 µL in the Reflotron System (Boehringer Mannheim, Germany). Only animals with fast glycemia levels above 180 mg/dL were included in the study. Diabetics with levels above 500 mg/dL or less than 180 mg/dL were excluded from the study (Fig. 1). In addition, samples for plasma glucose measurement were taken on the last day of experiments.

### Treatment with PPADS

The treatment was based on two IP injections of 12.5 mg/kg of PPADS in 0.1 mL of vehicle (0.9% sodium chloride), or 0.1 mL of vehicle, according to the corresponding animal group. The first injection was given at 9 weeks of diabetes and the second one at 24 weeks of diabetes.

### Animal study groups

#### Diabetic groups

Two diabetic groups were made of five diabetic rats each. Group 1 was treated with two IP doses of PPADS, while Group 2 received two IP injections of 0.1 mL of vehicle.

#### Non-diabetic groups

The two control groups included age-matched non-diabetic rats. Each group of rats received an IP injection of 0.1 mL of vehicle solution at the beginning of the study. Then, according to the treatment time of diabetic rats, five control rats received IP doses of PPADS and five rats were treated with IP injections of 0.1 mL of vehicle solution (PPADS and vehicle control groups).

Diabetic animals and non-diabetic animals were sacrificed at 34 weeks of diabetes or at a matching age, respectively. Animals were handled according to the ARVO Statement for the use of animals in ophthalmic research.

### Immunohistochemical analyses

The eye was removed and fixed for 48 h in 4% paraformaldehyde (Sigma-Aldrich, St Louis, MO). It was then immersed in 4 concentrations of glucose (5% overnight, 7.5, 10 and 20%) for cryoprotection and interlocked with resin. Ten-micron sections were obtained and fixed on poly-l-lisine-treated glass slides (Shandon AS325 Retraction). For immunohistochemistry, the sections were first incubated with 1 µg/µL of biotinylated goat anti-mouse IgG, then in avidin-biotin peroxidase complex Kit and finally in 3,3′-diaminobenzidine (DAB)/nickel solution. The P2X2 immunoreactivity was analysed using the P2X2 antibody (sc-25693 Santa Cruz Biotechnology, CA), P2Y2 by the P2Y2 antibody (sc-15209 Santa Cruz Biotechnology, CA) and VEGF-A immunoreactivity was examined with VEGF-A antibody (sc-1836 Santa Cruz Biotechnology, CA).

### Immunofluorescence analyses

Axial sections were revealed using 1 µg/µL of mouse anti-goat secondary antibody with fluorescein. Immunofluorescent analysis was done using the Eclipse Nikon Microscope (Tokyo, Japan). The GFAP expression was studied using 2 µg/µL of mouse anti-GFAP (BIOGENEX, 4600 Norris Canyon Road, San Ramon, CA, USA), while the P2Y2 was analysed using 2 µg/µL of goat anti-P2Y2 antibody (sc-15209 Santa Cruz Biotechnology, CA).

### Western blot (WB)

Isolated retinas were rinsed in the lysis buffer (5 mM Tris-HCl pH 6.8, 2 mM MgCl_2_, 2 mM EDTA, 65 mM NaCl, 1% Triton X-100) and cocktail protease inhibitor (Sigma-Aldrich, St. Louis MO, USA). Protein concentration was determined according to Bradford method [18]. Total protein (10 μg per well) was used in an electrophoresis on a 12% SDS-polyacrylamide gel and blotted onto nitrocellulose. The blot was incubated with primary antibody for 1.5 h at room temperature, washed three times with Trizma (buffer pH 7.4 with 0.1% of Tween 20) and further incubated in a secondary antibody for 1 h at room temperature. The bands were visualized using the enhanced chemiluminescence detection system (ECL, Amersham, Arlington Heights, IL, USA). The dilution for each antibody was 1:1000 P2Y2 (sc-15209 Santa Cruz Biotechnology, CA), 1/1000 P2X2 (sc-25693 Santa Cruz Biotechnology, CA), 1:700 VEGF (sc-1836 Santa Cruz Biotechnology, CA), and 1:700 actin (sc-1615 Santa Cruz Biotechnology, CA). The secondary antibody used was goat conjugated to a streptoavidin-peroxidase enzyme, and dilution was 1:10,000. All antibody dilutions were made in Trizma-base 0,01 M pH 7,4 with 1% Bovine Serum Albumin (BSA).

### Histological examination

Rats were anaesthetized with an IP injection of 350 mg/kg of chloral hydrate. The eye was removed and fixed in 4% paraformaldehyde (Sigma-Aldrich, St Louis, MO). Animals were sacrificed with an overdose of chloral hydrate. The eye was left for fixation in 4% paraformaldehyde for 1 day. Afterwards, it was immersed in increasing concentration of glucose (5% overnight, 7.5, 10 and 20%) and interlocked with resin. Ten-micron cryosections were obtained (Shandon AS325 Retraction) and stained with Hematoxylin and eosin (H&E) as well as Periodic acid-schiff (PAS) and eosin for microscopic examination using an Eclipse Nikon E800 Microscope (Tokyo, Japan).

### Retinal ganglion cell counting and retinal thickness

H&E stained cryosections were analyzed to count RGCs. Briefly, retinas were divided on seven sections including three fields in central retina, two fields in medial retina and two fields in peripheral retina at 40×. RGCs were counted in each field for each cryosection, results are showed like whole average for each cryosection for each group of animals (N = 5).

For retinal thickness, H&E cryosections were observed at 40× and 20 measures were taken, one every 300 µM, starting on the beginning of peripheral retina. Each measure started on Outer Nuclear Layer (ONL) and end at RGCs Layer. Results are showed like an average of the measures taken in each cryosection for each group (N = 5).

### Statistical analysis

Results in this work are expressed as a mean ± standard error of the mean, and were analyzed with ANOVA and Newman–Keuls multiple comparison post-test.

## Results

### Fast glycemia

Fast glycemia levels were normal (100 mg/dL or below) on the day prior to the intraperitoneal injection of STZ or vehicle. At day 2 post-injection fast glycemia levels of 95 mg/dL or lower and 200 mg/dL or higher were found in non-diabetic and diabetic animals, respectively.

Glucose levels were greater than 200 mg/dL in animals treated with STZ prior to injection with PPADS, whereas the control animals had values close to 100 mg/dL (Fig. 1a). After treatment with PPADS surprisingly, the glucose levels of the treated diabetics decreased relative to the untreated diabetics and the glucose levels in the treated controls increased compared to the untreated controls (Fig. 1b).

### P2Y2 immunoreactivity

#### Diabetic animals and controls without treatment

**Fig. 2:**
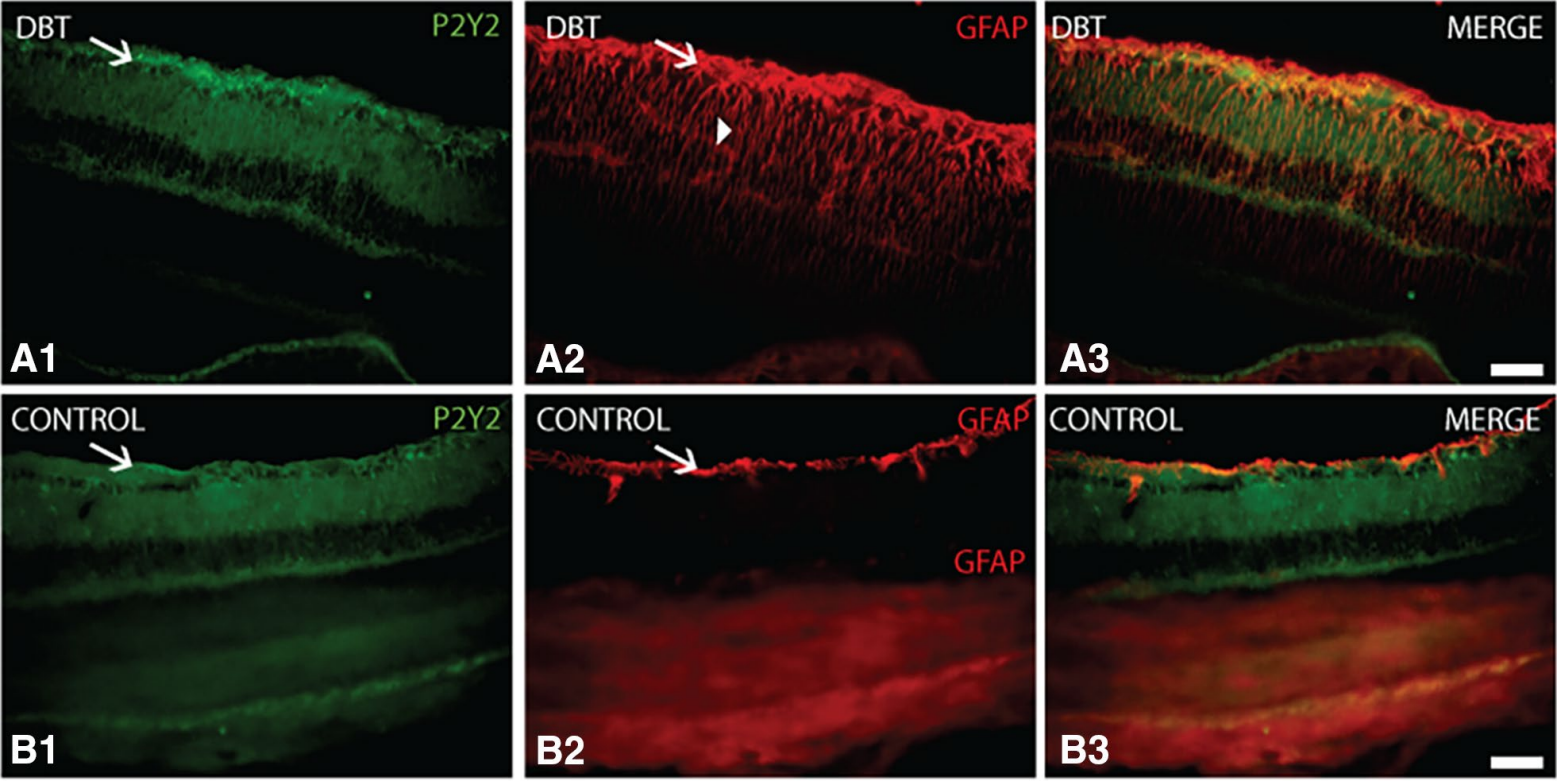
Retinal cross section. Immunofluorescent analyses of diabetic and control animals without purinergic receptors antagonist treatment. **A** Diabetic group (34 weeks after STZ IP injection). **B** Control group. P2Y2r immunoreactivity was seen in the fiber layer of diabetic and control animals (arrow-A1 and B1). In the diabetic group staining had a larger extension. Positive immunoreactivity of GFAP was observed in glial cells of diabetics and controls (arrow A2 and B2). In diabetic animals immunoreactivity was much more extended and some GFAP positive cells showed a morphology similar to that seen in Müller Cells (arrow head-A2). Co-expression of P2Y2 and GFAP in diabetics and controls had different patterns. In diabetic rats co-expression of P2Y2 and GFAP was seen in the fiber layer and in glial cells extending to the photoreceptor layer (A3), while in controls the co-expression was slightly observed in the fiber layer (B3)

P2Y2 immunoreactivity was seen in the ganglion cell layer of diabetic and control animals. Diabetic group staining had a larger extension and was also observed at the photoreceptor inner segment (Fig. 2A1). As expected, GFAP staining was greater in the diabetic groups than control (Fig. 2A2, B2). Merge of P2Y2 and GFAP in diabetics and controls had similar patterns (Fig. 2A3, B3). In summary, the fiber layer, glial cells and the photoreceptor layer P2Y2 expression was seen in diabetic rats, while in controls the P2Y2 expression was slightly observed in the fiber layer. Same results were observed for immunofluorescence and immunohistochemistry (not shown).

#### Immunohistochemical analysis of diabetic animals and controls treated with PPADS

**Fig. 3:**
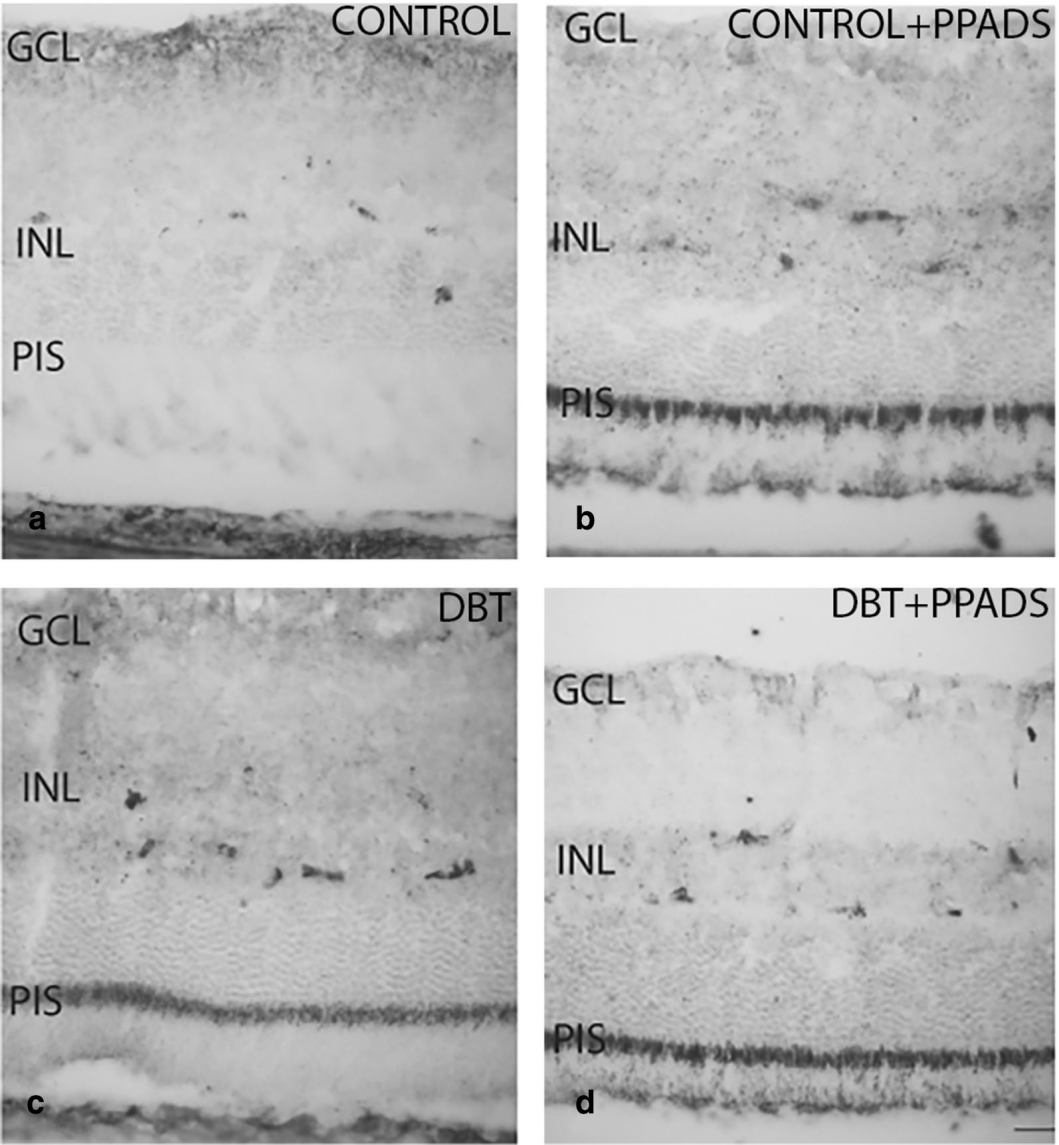
Retinal cross-sections. P2Y2 immunohistochemistry in diabetic and control animals treated with purinergic receptors antagonist (PPADS). **a** and **b** represent control groups. **c** and **d** represent diabetic groups. **b** and **d** groups were treated with intraperitoneal injection of group with PPADS. P2Y2r immunostaining was seen in the photoreceptor inner segment in all diabetics and in controls treated with purinergic receptors antagonist. *GCL* ganglion cells layer; *INL* Inner nuclear layer; *PIS* photoreceptor inner segment; *DBT* diabetic; *PPADS* animals treated with pyridoxalphosphate-6-azophenyl-2′,4′-disulfonic acid

The immunohistochemical pattern of P2Y2 was quite similar in retinas of diabetic animals either treated or not treated with PPADS (Fig. 3c, d). The pattern observed in diabetics was different from that found in control without treatment (Fig. 3a, c). Also, both diabetic groups there were a positive immunoreactivity of the photoreceptor inner segment, and this was also seen among control animals treated with a purinergic antagonist (Fig. 3b–d).

### P2X2 immunoreactivity

#### Diabetic animals and controls without treatment

**Fig. 4:**
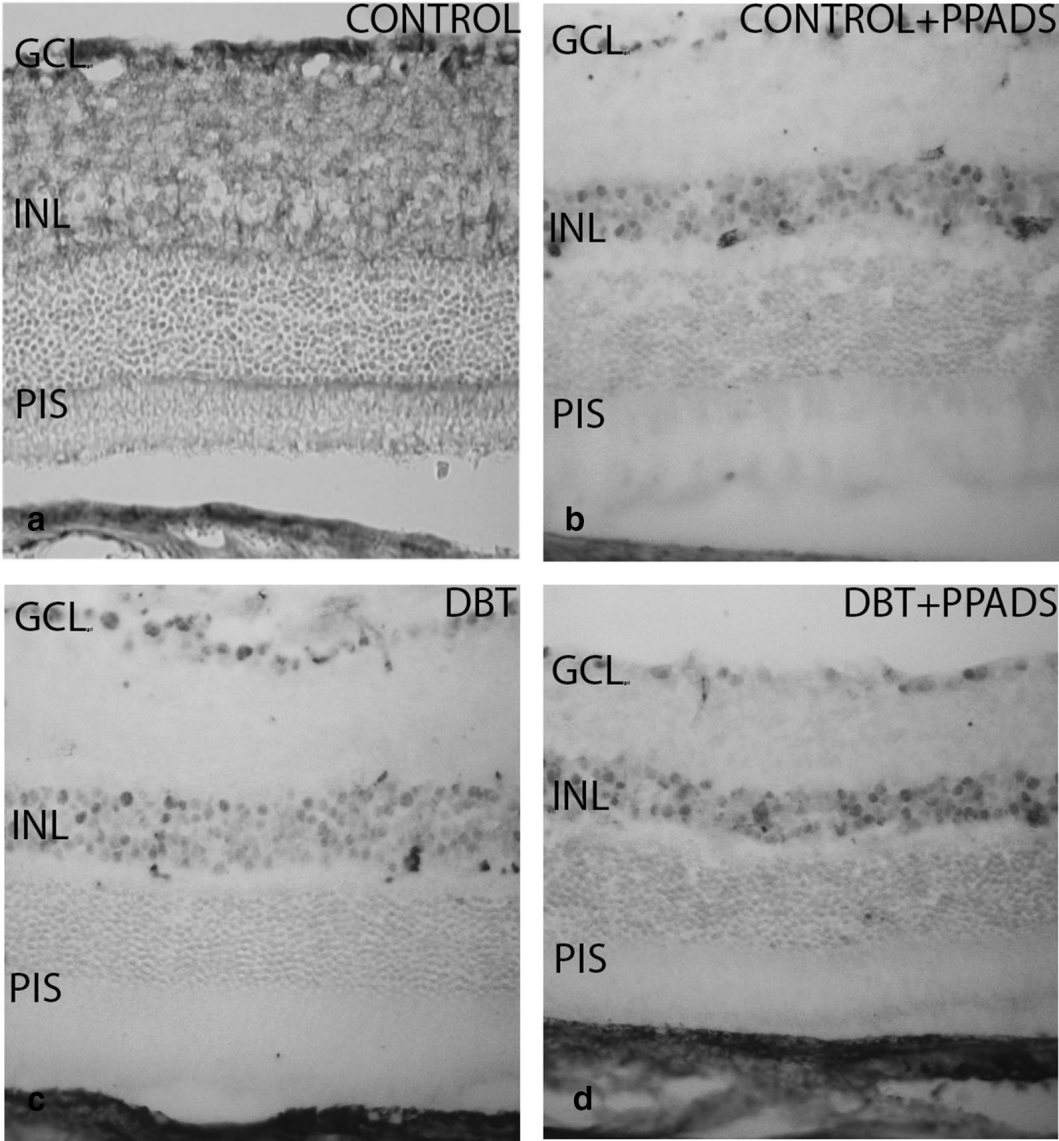
Retinal cross-sections. P2X2 immunohistochemistry in diabetic and control animals treated with purinergic receptors antagonist (PPADS). **a** and **b** Represent control groups. **c** and **d** Represent diabetic groups. **b** and **d** Groups were treated with intraperitoneal injection of PPADS. P2X2r immunostaining was seen in the photoreceptor inner segment and outer nuclear layer of controls without treatment. In the remaining animal groups immunoreactivity was found in the inner nuclear layer. *GCL* ganglion cells layer; *INL* Inner nuclear layer; *PIS* photoreceptor inner segment; *DBT* diabetic; *PPADS* animals treated with pyridoxalphosphate-6-azophenyl-2′,4′-disulfonic acid

P2X2 immunoreactivity was observed in the ganglion cell layer of diabetic and control animals. However, staining was larger in the control group and it was seen in the ganglion cell layer, inner plexiform, outer nuclear and photoreceptor layers (Fig. 4a).

#### Immunohistochemical analysis of diabetic animals and controls treated with PPADS

The immunohistochemical pattern of P2X2 was similar in retinas of diabetic animals treated or not treated with PPADS (Fig. 4b, d). In both groups there was a positive immunoreactivity in the ganglion cell and inner nuclear layers. Although the immunoreactivity of P2X2 was reduced in treated groups respect to control group, the difference was more pronounced than that found compared to the diabetic group (Fig. 4a–d).

### VEGF-A immunoreactivity

**Fig. 5:**
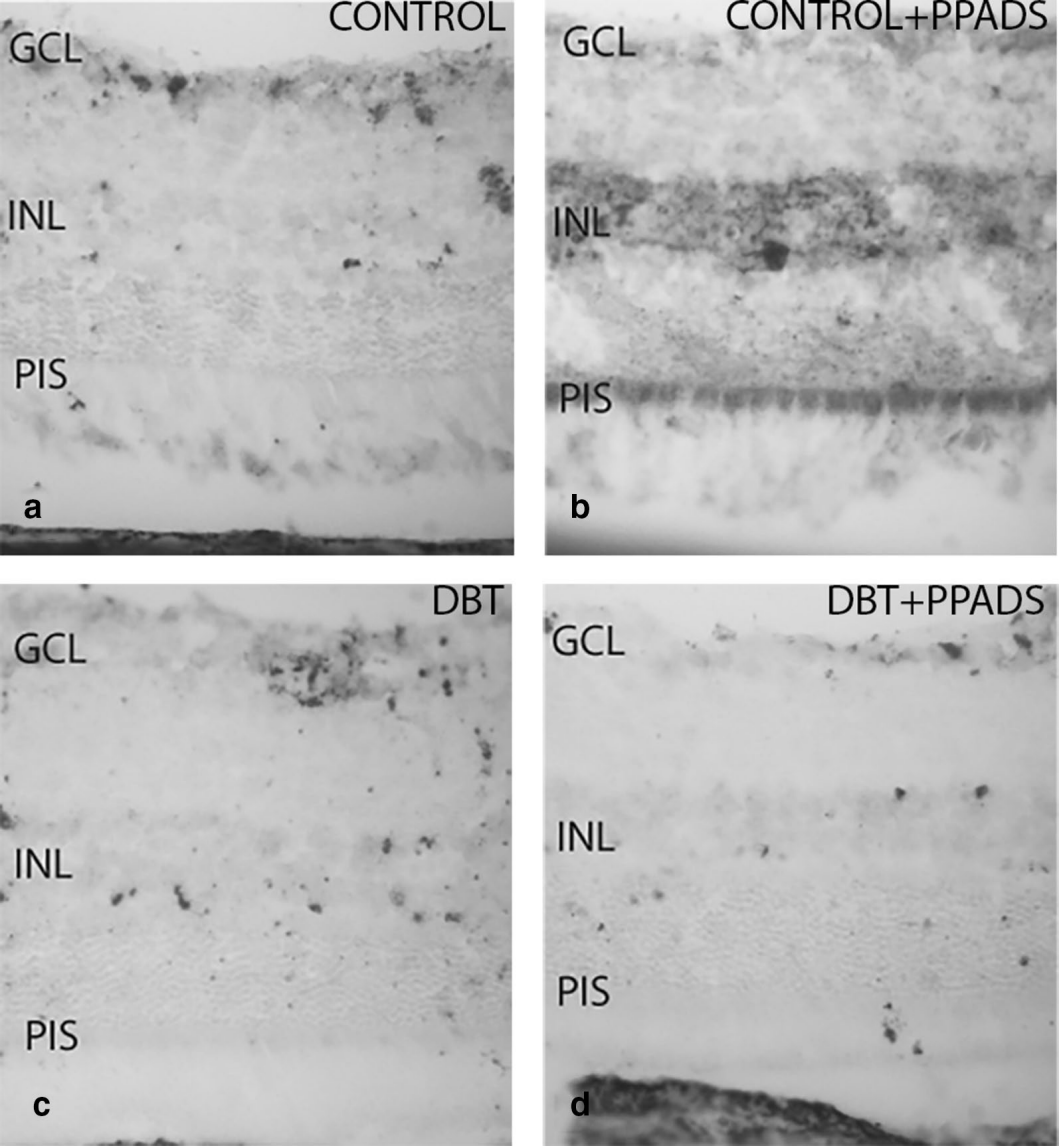
Immunohistochemical analyses of VEGF-A. Immunostaining of VEGF-A was found in two different patterns. **b **Controls with PPADS treatments showed a positive immunoreactivity in GCL, INL and PIS. **a **Controls without treatment and **c, d **diabetic animals showed staining in structures which may be small vessels (arrows). *GCL* ganglion cells layer; *INL* Inner nuclear layer; *PIS* photoreceptor inner segment; *DBT* diabetic; *PPADS* animals treated with pyridoxalphosphate-6-azophenyl-2′,4′-disulfonic acid

The pattern was found to be different among control and diabetic groups (Fig. 5a, c, d). The extension of immunoreactivity was larger in diabetics than in controls. VEGF-A immunoreactivity was seen in small vessels, fiber layer, INL, and in RPE of all diabetics. However, diabetic animals treated with, PPADS disclosed a much more extended staining than diabetics without treatment (Fig. 5c, d). In control animals without treatment the immunostaining of VEGF-A was observed in vessels, INL and the photoreceptor outer segment (Fig. 5a). The pattern observed in control animals treated with PPADS consisted of positive immunoreactivity of VEGF-A in the GCL, INL and PIS (Fig. 5b, d).

### P2X2, P2Y2 and VEGF protein expressions

**Fig. 6:**
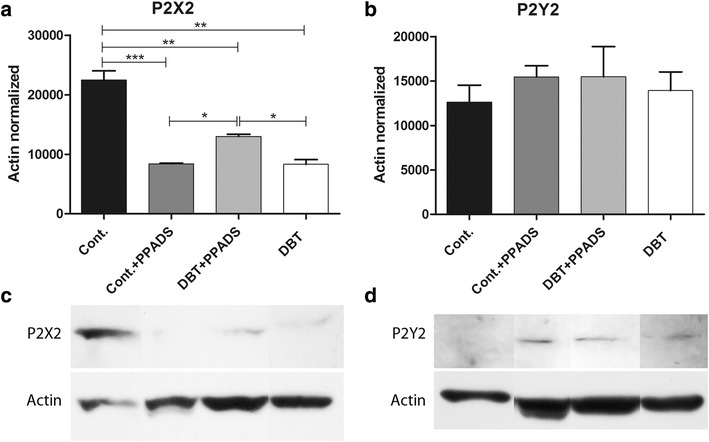
Western blot analyses of P2X2 and P2Y2. **a** P2X2 densitometry quantification, lower expression was seen in the diabetic and treated groups. **b** P2Y2 densitometry quantification, non-significant differences were found between groups. **c** P2X2 immunoblot. **d** P2Y2 immunoblot. Diabetic animals treated with PPADS showed higher P2X2 expression levels compared with non-treated diabetic group. *Cont.* control; *PPADS* pyridoxalphosphate-6-azophenyl-2′,4′-disulfonic acid; *DBT* diabetic (*p<0.05; **p<0.01; ***p<0.001)

Western blot analyses revealed a significantly lower expression of P2X2 in diabetics without treatment than control norm glycemic group. Diabetics treated with the PPADS had higher expression of P2X2 compared to diabetic rats without treatment (Fig. 6a, c). Non- significant differences were found for P2Y2 protein expression between experimental groups (Fig. 6b, d).

**Fig. 7:**
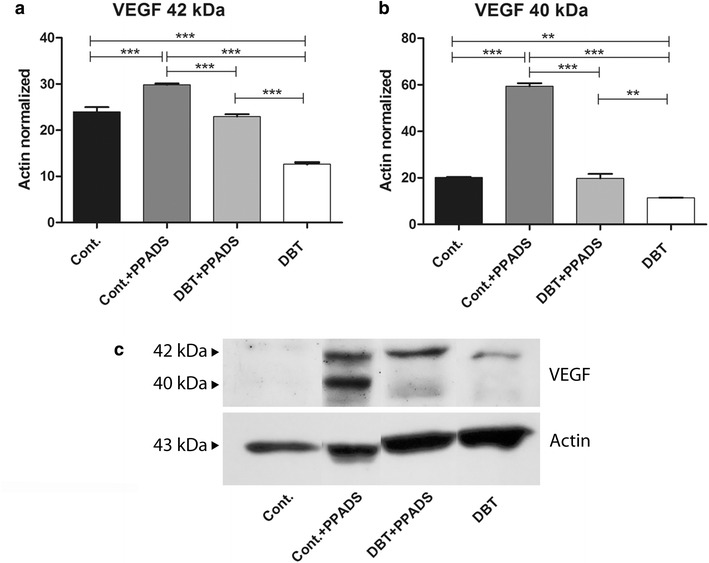
Western blot analyses of VEGF-A. **a** 42 KDa band. The expression was lower in diabetic group compared to control group or PPADS groups. **b** 40 KDa band. The expression profile was similar than 42 kDa band. **c** Inmunoblot. *Cont.* control; *PPADS* pyridoxalphosphate-6-azophenyl-2′,4′-disulfonic acid; *DBT* diabetic (*p<0.05,**p<0.01, ***p<0.01)

We identified two western blot banding patterns for VEGF, one at 42 kDa and the other at 40 kDa. The protein expression of VEGF-A at 42 kDa was lower in diabetic and non-diabetic animals without treatment compared to non-diabetics treated with PPADS (Fig. 7a, c). Diabetic animals with PPADS treatment showed a similar expression of VEGF-A than non-diabetic controls without treatment. The expression of VEGF at 40 kDa, probably corresponding to the 165b dimmer, have a similar expression pattern than VEGF at 42 kDa (Fig. 7b, c). This is consistent with immunohistochemistry results where VEGF showed higher levels of protein expression in PPADS treated non-diabetic group (Fig. 5a).

### Retinal ganglion cell counting and retinal thickness

**Fig. 8:**
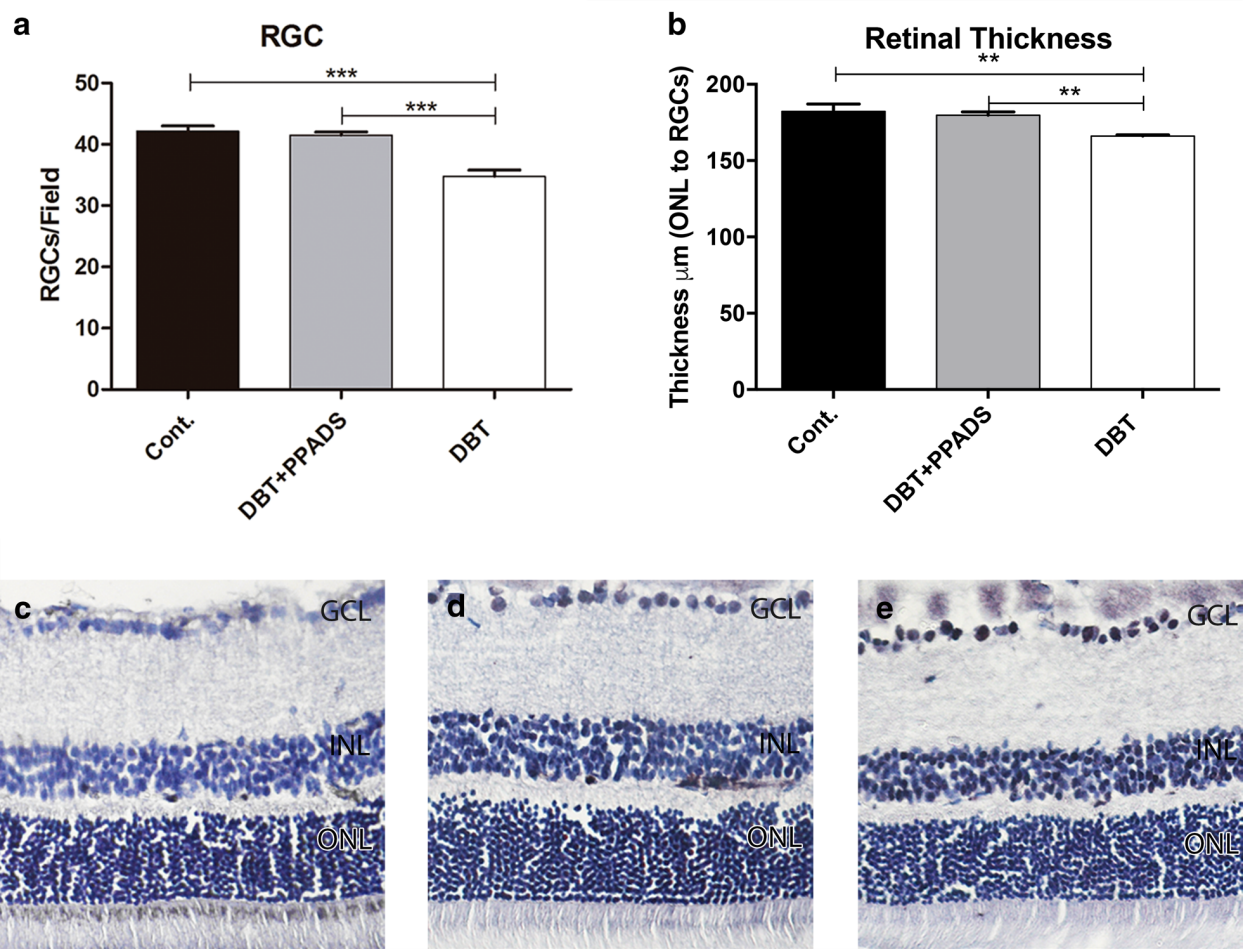
**a** Retinal ganglion cell counting. **b** Retinal thickness. **c** Cryosection stained with H&E for control group without treatment. **d** Cryosection stained with H&E for treated diabetic group. **e** Cryosection stained with H&E for diabetic group. *Cont.* control; *PPADS* pyridoxalphosphate-6-azophenyl-2′,4′-disulfonic acid; *DBT* diabetic (*p<0.05,**p<0.01, ***p<0.01)

The number of RGCs in non-treated diabetic animals were lower respect to non-diabetic animals. Diabetic animals treated with PPADS recovered normal levels of density of RGCs. The same pattern was reflected with retinal thickness (Fig. 8).

## Discussion

We carried out a research study to evaluate the role of P2Y2 and P2X2 in the retina of a rat model of diabetes. The study included diabetic and non-diabetic animals divided into subgroups either treated with an intraperitoneal injection of vehicle or a purinergic receptor antagonist. We found a down-regulation of P2X2 in the retina of treated and untreated diabetic animals. However, with PPADS treatment we observed a higher protein expression than in non-treated animals, suggesting a slightly recovery. It is known that purinergic receptors may regulate cell growth in an ATP-dependent manner. In this study, we demonstrated that the use of a purinergic antagonist in diabetic animals induced changes in the protein expression of VEGF-A. VEGF-A was downregulated in diabetic animals, although, in treated animals had the same levels of expression than non-treated control group. Our results suggest a relationship between this neurotrophic and pro-inflammatory agent and the purinergic pathway but further experiments are required.

Purinergic signaling seems to be an intermediary in the communication between the retina and the RPE [37]. In this study, P2Y2 expression at the photoreceptor outer segment was observed in all groups of diabetic animals and in the group of non-diabetic animals treated with the purinergic antagonist. This finding was not seen in non-diabetic animals. As we already know, PPADS has a selective effect on P2 receptors, so these results suggest that PPADS have an effect on the P2Y2 expression at photoreceptors level. Furthermore, diabetic animals have a higher glucose metabolism and an increase in ATP production. Besides, ATP may also activate P2 receptors of neighboring retinal neurons, such as photoreceptors, amacrine cells, and ganglion cells [5, 6, 38]; and we think that these changes lead to an augmented purinergic signaling as we have seen in the present study. On the other hand, non-diabetic animals (not treated with purinergic antagonist) showed lower P2Y2 expression, a fact that supports our hypothesis.

The neuroprotective role of P2Y2 and P2X2 has been suggested by other researchers [32, 39–42]. Up-regulation of P2Y2 and agonists enhanced this effect in astrocytes [43] and a selective antagonist (AR-C118925) could inhibit glial activation [44]. In this study, diabetic animals treated with PPADS showed a higher protein expression of VEGF-A, known to have a neurotrophic effect [34, 35]. These findings support the idea of the neuroprotective role of P2X2. This situation was also reflected in RGC count and retinal thickness in our study.

Pancreatic cells contain stores of ATP as well as purinergic receptors [19, 45]. It is well established that ATP and ADP induce insulin secretion in presence of glucose, The disturbance of the purinergic pathway caused by PPADS might result in changes of insulin and glucagon secretion [45]. The P2X receptor might elicit insulin secretion even in low glucose concentration. We think that this would explain the unexpected elevated levels of fast glycemia observed in non-diabetic animals after two treatment cycles of purinergic antagonists. Moreover, we think this is an interesting finding that warrants further investigation.

The P2Y2 nucleotide receptor seems to inhibit trauma-induced death of astrocytic cells [46]. This protective effect may be achieved through agonists as ATP. Besides, a previous study reported a new pathway for neuronal survival through the activation of P2Y2 receptor. It is feasible the existence of interacting systems –extracellular nucleotides/P2Y2 receptors and neurotrophin/TrkA—to sustain the survival of neurons [47]. It is well established that diabetes increases cell death of retinal ganglion cells by apoptosis [48]. This has probably occurred in the current study, where we observed an increased RGC loss and a reduced retinal thickness in the diabetic group respect to controls and treated diabetic animals.

In non-diabetic treated animals, we found a higher protein expression of VEGF-A at 42 KDa compared to non-diabetics without treatment and diabetics with treatment. Another study has proposed that VEGF receptor can be activated in the absence of VEGF. P2YR-VEGFR2 could interact resulting in a signal transduction that is a critical determinant of vascular homeostasis and tumor-mediated angiogenesis [49]. Besides, it should be noted that VEGF-A affects the development of new vessels (angiogenesis) [50] as well as the vascular maintenance (endothelial cells survival and vascular permeability) and the neurotrophism in a physiological condition [34, 35, 51].

In the present study, non-diabetic animals treated with purinergic antagonists showed two bands of VEGF-A expression, one at 42 KD and the other at 40 KDa, forming a 2-band pattern. It is known that VEGF-A has isoforms depending of the splicing [52]. Most VEGF-A producing cells appear to preferentially express VEGF-A121, VEGFA165 and VEGF-A189, and murine counterparts are VEGF-A 120, 164 and 188, respectively. VEGF-A 165 is primarily isoform [53]. Its molecular weight commonly ranges from 38.2 to 45 [54]. In our study PPADS might have hindered ATP binding to P2X2 causing an increase in free ATP and facilitating the binding of ATP to VEGF-A. Binding of ATP to vascular endothelial growth factor isoform VEGF-A165 is well-known [53]. Besides, an identical isoform can have distinct activities at different anatomical sites, suggesting that the microenvironment of different tissues can dictate VEGF-A function [55]. We believe that the difference in VEGF-A expression and the 2-band pattern found is due to an altered imbalance of signaling in the purinergic pathway. Anyhow, new studies need to be undertaken to learn the meaning of these facts.

## Conclusions

In our experimental study, we have observed a decrease of P2X2 expression in the retina of diabetic rats. In these animals, the use of PPADS, a non-specific antagonist for purinergic receptors, upregulates the protein expression of molecules involved in cell survival and inflammation. We hope our study has shed light to mechanisms of diabetic retinopathy associated with the purinergic pathway. More extensive studies are required to identify the exact role of purinergic signaling in diabetic retinopathy development.
